# Genome-Wide Analysis, Expression Profile, and Characterization of the Acid Invertase Gene Family in Pepper

**DOI:** 10.3390/ijms20010015

**Published:** 2018-12-20

**Authors:** Long-Bin Shen, Yu-Ling Qin, Zhi-Qiang Qi, Yu Niu, Zi-Ji Liu, Wei-Xia Liu, Huang He, Zhen-Mu Cao, Yan Yang

**Affiliations:** Tropical Crops Genetic Resources Institute, Chinese Academy of Tropical Agricultural Sciences/Key Laboratory of Crop Gene Resources and Germplasm Enhancement in Southern China, Ministry of Agriculture, Danzhou 571737, China; LongbinShen@catas.cn (L.-B.S.); yulingqin2017@catas.cn (Y.-L.Q.); ZhiqiangQi@catas.cn (Z.-Q.Q.); niuyu@catas.cn (Y.N.); liuziji1982@catas.cn (Z.-J.L.); weixialiu@catas.cn (W.-X.L.); HuangHe@catas.cn (H.H.)

**Keywords:** acid invertase, pepper, gene expression, enzymatic activity

## Abstract

Catalytic decomposition of sucrose by acid invertases (AINVs) under acidic conditions plays an important role in the development of sink organs in plants. To reveal the function of AINVs in the development of pepper fruits, nine *AINV* genes of pepper were identified. Protein sequencing and phylogenetic analysis revealed that the CaAINV family may be divided into cell wall invertases (CaCWINV1–7) and vacuolar invertases (CaVINV1–2). CaAINVs contain conserved regions and protein structures typical of the AINVs in other plants. Gene expression profiling indicated that *CaCWINV2* and *CaVINV1* were highly expressed in reproductive organs but differed in expression pattern. *CaCWINV2* was mainly expressed in buds and flowers, while *CaVINV1* was expressed in developmental stages, such as the post-breaker stage. Furthermore, invertase activity of CaCWINV2 and CaVINV1 was identified via functional complementation in an invertase-deficient yeast. Optimum pH for CaCWINV2 and CaVINV1 was found to be 4.0 and 4.5, respectively. Gene expression and enzymatic activity of CaCWINV2 and CaVINV1 indicate that these AINV enzymes may be pivotal for sucrose hydrolysis in the reproductive organs of pepper.

## 1. Introduction

Pepper (*Capsicum annuum* L.) is a vegetable crop of worldwide importance. Pepper fruit, which is used extensively as a seasoning, is rich in natural pigments, vitamin C, and carotenoids [1]. Abortion of flowers/fruits, which affects the yield of peppers, is a serious problem faced by pepper cultivators. In severe cases, it may lead to a total loss of harvest. Pepper flowers and fruits function as a typical sink organ. The abortion of flowers/fruits is related to the source and sink strength of the pepper plant [2]. It has been reported that acid invertase (AINV) and sucrose synthase activities may be associated with sink activity of pepper [3]. Acid invertases catalyze the irreversible hydrolysis of sucrose into glucose and fructose in flowers/fruits, and thus plays an important role in the development of pepper flowers/fruits [4]. The activity of acid invertase varies with the stage of pollen development. Its activity, which is low during the early stages, increases sharply 2 days before anthesis and remains elevated [5]. By contrast, acid invertase activity increases during pepper fruit ripening, causing the accumulation of hexose sugars. These studies indicate that acid invertase may play an important role in the growth and development of pepper reproductive organs. 

In higher plants, acid invertases, which are systematically termed β-fructofuranosidases, exhibit an acidic pH optimum of between 4.5 to 5.0, and may be distinguished by their subcellular localization [6]. Cell wall invertase (CWINV) is localized within the cell wall, and vacuolar invertase (VINV) in the vacuole. Acid invertases are encoded by a family of genes originating from respiratory eukaryotes and aerobic bacteria [7]. It is believed that vacuolar invertases evolved from cell wall invertases. During the process of plant evolution, the N-terminal signal peptide of cell wall invertase may have mutated to transmembrane domain (TMD), a basic region (BR) motif, and a YXXØ motif, and formed a complex N-terminal propeptide (NTPP) region, enabling vacuolar invertase to travel to the vacuole [8,9]. Three conserved motif-N**D**PNG (β-fructosidase motif), R**D**P, and W**EC**P(V)D were found in all acid invertases of plant. Conserved residues (underline and bold face) of these motifs reportedly play a crucial function in the hydrolysis of sucrose into glucose and fructose [8,10]. Three amino acids (DPN) of the β-fructosidase motif are encode by 9 bp long exon, which is the smallest known to function among plant species [11]. More recently, acid invertase family genes have been identified in the genomes of species, such as rice [8] and poplar [7]. However, the acid invertase genes of pepper have not been reported. In this study, all of the acid invertases were analyzed, based on the available sequences of the pepper genome database. The evolutionary relationships, exon–intron structures, motif distributions, subcellular localizations, and three-dimensional (3D) structures of CaAINV proteins were investigated. Temporal and spatial expression patterns of *CaAINVs* were analyzed via published RNA-sequence data. Finally, acid invertase activity of CaCWINV2 and CaVINV1 was investigated through functional complementation experiments using the invertase-deficient yeast strain, SEY2102. These results contribute to a further understanding of the role of acid invertases in sucrose metabolism during the development of pepper.

## 2. Results

### 2.1. Identification and Characterization of CaAINV Genes in Pepper

**Figure 1:**
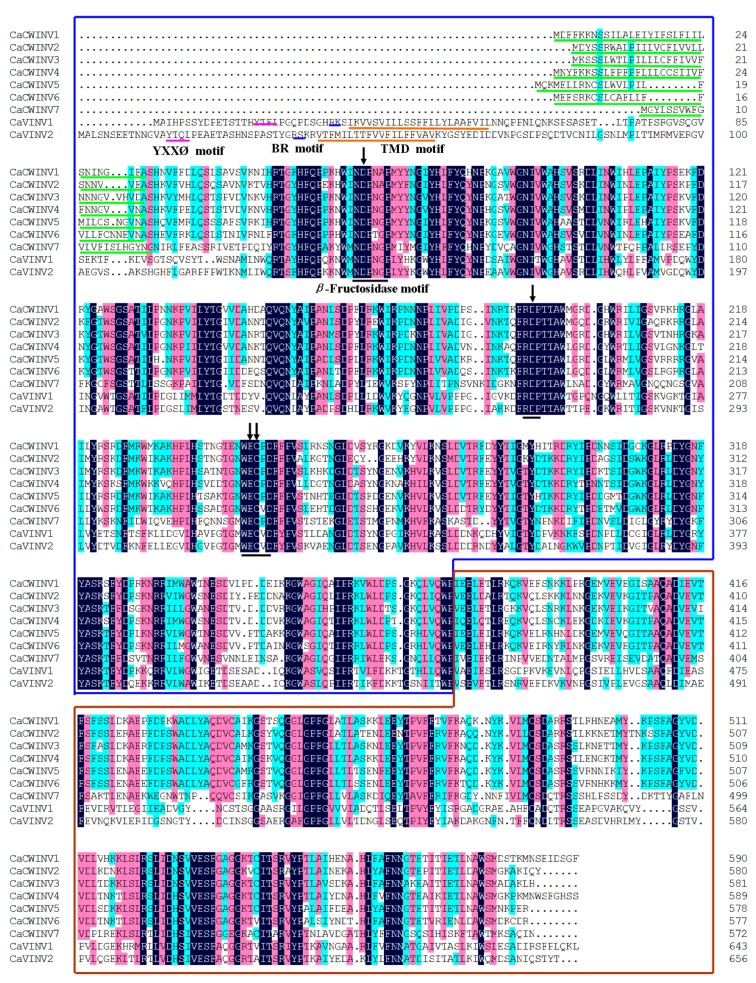
Alignment of deduced amino acid sequences of the nine pepper acid invertases. Dark-blue shading, pink shading, and light blue shading reflect 100%, 75%, and 50% amino acid residues conservation, respectively. Catalytic residues are depicted by black arrows. Green lines indicate the predicted signal peptide. Black lines indicate the conserved motif-NDPNG (β-fructosidase motif), RDP and WECP(V)D. Brown lines indicate the predicted transmembrane domain (TMD). The blue lines indicate basic region (BR) motif. The pink lines indicate YXXØ motif.

*Arabidopsis* acid invertase genes were subjected to a Basic Local Alignment Search Tool (BLASTN) search against the pepper genome database in order to identify AINV genes. A total of nine acid invertase family genes were identified, of which seven members were cell wall invertases, named CaCWINV1 to 7, and two were vacuolar invertases, named as CaVINV1 and 2. Full-length coding sequences of the *CaAINV* genes ranged from 1719 bp (*CaCWINV7*) to 1971 bp (*CaVINV2*). The size of the deduced CaAINV proteins varied between 572 and 656 amino acids (aa), with an average of 596 aa. Their molecular weight (*M*_w_) varied from 64.36 to 73.29 kDa, and the theoretical pI of these genes ranged from 5.51 to 9.60 (Table 1). Alignment analysis of the deduced amino acids showed that the CaAINVs share 48.25% to 80.38% identities among all the family genes. Three conserved motif-NDPNG (β-fructosidase motif), RDP and WECP(V)D were found in all identified CaAINV proteins. All CaCWINVs contain a putative signal peptide with a predicted cleavage site from 22 to 32 amino acids at the N-terminus (Figure 1), where these proteins were putatively localized to the extracellular space according to TargetP 1.1 Server analysis. By contrast, CaVINV1 and CaVINV2 contained a transmembrane domain (TMD), a basic region (BR) motif, and a YXXØ motif in the N-terminal sequence. These characteristic features suggested that they were members of the acid invertase gene family.

### 2.2. Phylogenetic Analysis of Acid Invertase Genes in Pepper

**Figure 2:**
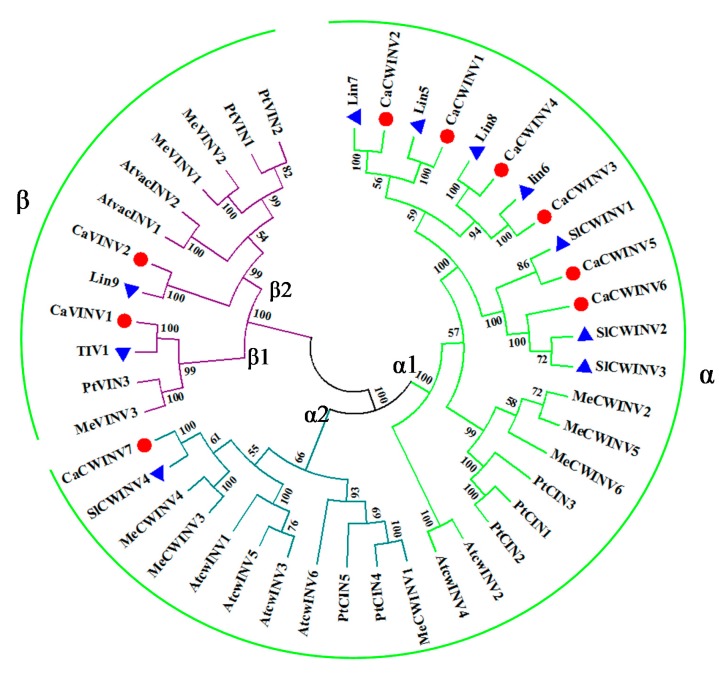
Phylogenetic analysis of acid invertase (AINV) proteins from pepper, cassava, Arabidopsis, and poplar. The phylogenetic tree was constructed via the Neighbor-Joining method (1000 bootstrap replicates) using Molecular Evolutionary Genetics Analysis 7.0 software. Red and blue dots indicate AINVs from pepper and tomato, respectively.

For phylogenetic analysis, the nine full-length CaAINVs identified in this study, as well as ten from tomato, eight from *Arabidopsis*, nine from cassava, and eight from poplar, were used. These proteins were classified into two major groups, α and β, based on the phylogenetic tree (Figure 2). Seven proteins containing a putative signal peptide, CaAINV1, 2, 3, 4, 5, 6 and 7, were clustered together in the α group. Proteins containing a complex N-terminal propeptide (NTPP) region, CaVINV1 and 2 were clustered in the second major group, termed the β group. In the α group, CaCWINV1, 2, 3, 4, 5, and 6 had a close relationship with Lin5, 6, 7, 8 and SlCWINV1, 2, 3, where these genes were categorized as the α1 group. CaCWINV7 and SlCWINV4 formed a clade and were grouped as the α2 group. In the β group, CaVINV1 had a close relationship with TIV1, and these were categorized as the β1 group. CaVINV2 and Lin9 had a close relationship and were grouped as β2. All CaAINV proteins have a close relationship with AINV proteins from tomato (Figure 2). The two species belong to *Solanaceae Juss*.

### 2.3. Structural Analysis and Chromosomal Distribution of CaAINV Family Genes

**Figure 3:**
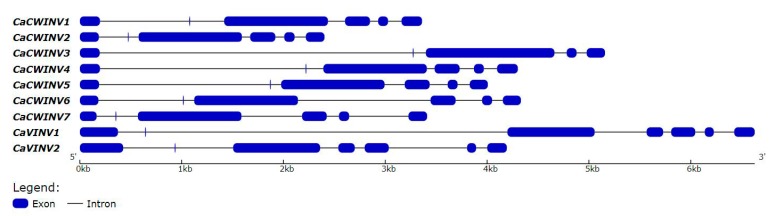
Exon–intron structure of the seven *CaAINVs* in pepper. Introns are depicted by black lines, exons are depicted by blue boxes.

In order to gain further insight into the evolutionary relationships between *CaAINVs*, the structures of these genes were analyzed by aligning the genomic and coding sequence (CDS) regions for each *CaAINV* gene. The number of exons in *CaAINV* genes ranged from five to seven (Figure 3). Most cell wall invertase genes, except CaCWINV3, contained six exons. Both vacuolar invertase genes contained seven exons. The second exon in all CaAINVs was a mini exon, located in the β-fructosidase motif (NDPNG) which encoded three amino acids. The first exon in CaCWINVs was shorter than CaVINVs.

**Figure 4:**
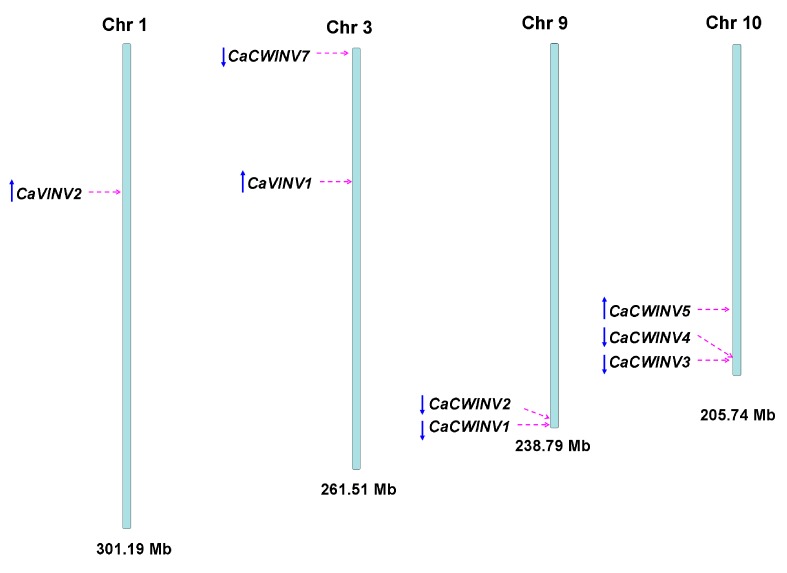
Chromosomal localization of *CaAINV* genes from pepper. The positions of the *CaAINV* genes are depicted by red arrows. The gene orientation is depicted by blue arrows.

The chromosomal distribution of pepper *CaAINV* genes was analyzed in order to gain an insight into their evolution. Results mapped the nine *CaAINV* genes to four chromosomes of the pepper genome (Figure 4). Tandem duplication events were detected in *CaCWINV* genes. Gene pairs of *CaCWINV1*/*2* and *CaCWINV3*/*4* had similar orientation and tandem duplications on chromosomes 1 and 10, respectively. *CaVINV1* was present on chromosome 1, while *CaVINV2* was mapped to chromosome 3 with similar orientation.

### 2.4. Motif Distribution in CaAINV Proteins

**Figure 5:**
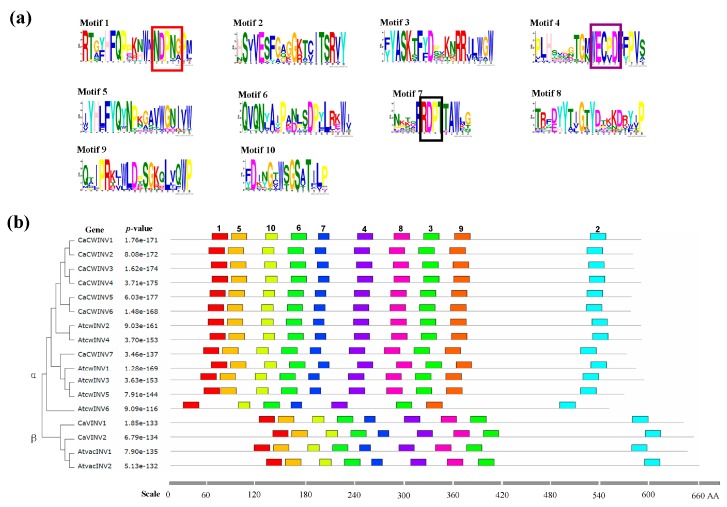
The motifs of AINV proteins from pepper and *Arabidopsis* according to phylogenetic relationships. (**a**) The ten motif sequences identified by Multiple Em for Motif Elicitation (MEME). The conserved motifs-NDPNG, RDP, and WECP(V)D are depicted by red, black, and purple boxes, respectively. (**b**) Motif distribution in AINVs. The phylogenetic relationship tree of CaAINVs and AtAINVs was constructed using Muscle and MEGA7 software. Gray lines depict non-conserved sequences, and each motif is depicted by a colored box. The length of the motifs in each protein is shown proportionally.

In order to further clarify the evolutionary relationship between CaAINV proteins, putative motifs of pepper and tomato AINV proteins were analyzed based on their evolutionary tree. Ten distinct motifs were identified. Most motifs classified as α or β CaAINV were similar. However, the non-conserved sequences at the N-terminus in the α group were longer, compared to the β group. The distribution of these motifs in CaAINVs and AtAINVs was similar (Figure 5). Motifs 1, 2, 3, 4, 6, 7, and 10 were widely distributed in all the AINV proteins that were analyzed. The conserved motifs-NDPNG, RDP, and WECP(V)D were localized in motifs 1, 4, and 7, respectively. In addition, motif 9 was specifically distributed in the α group, and AtCWINV6 lost motifs 5 and 8.

### 2.5. Three-Dimensional Structure of CaAINV Proteins

**Figure 6:**
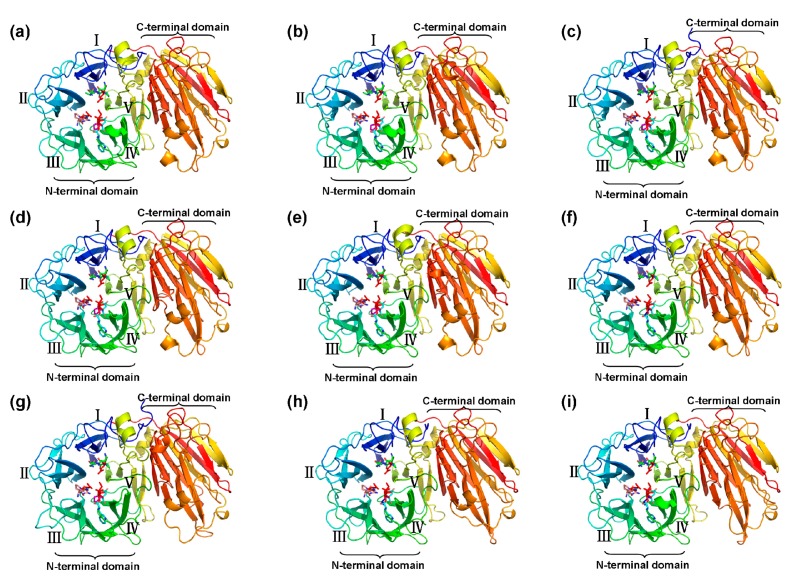
Predicted three-dimensional structure models of CaAINVs. (**a**) CaCWINV1; (**b**) CaCWINV2; (**c**) CaCWINV3; (**d**) CaCWINV4; (**e**) CaCWINV5; (**f**) CaCWINV6; (**g**) CaCWINV7; (**h**) CaVINV1; (**i**) CaVINV2. The five blades of the β-propeller module are indicated by roman numerals I–V, respectively. The motifs (NDPNG, RDP, and WECP(V)D) are depicted by sticks. The image was generated using the PyMOL program (Schrödinger, Inc., New York, NY, USA).

**Figure 7:**
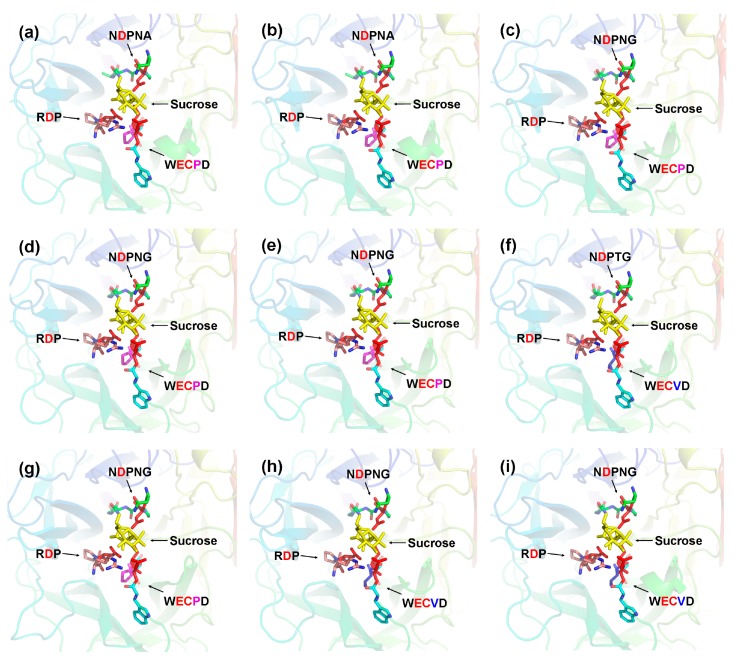
Predicted catalytic residues of CaAINV1 to 6 with sucrose molecules. (**a**) CaCWINV1; (**b**) CaCWINV2; (**c**) CaCWINV3; (**d**) CaCWINV4; (**e**) CaCWINV5; (**f**) CaCWINV6; (**g**) CaCWINV7; (**h**) CaVINV1; (**i**) CaVINV2. Yellow stick structures indicate sucrose molecules. The motifs (NDPNG, RDP, and WECP(V)D) are depicted by colored sticks. Red stick structures depict catalytic residues. The image was generated using the PyMOL program (Schrödinger, Inc., New York, NY, USA).

The three-dimensional (3D) structure of CaAINVs were modeled on the X-ray structure of AtcwINV1 from *Arabidopsis* (Protein Databank ID 2AC1, gene accession code At3g13790) using the Swiss-Model. AtcwINV1 shared 58.97%, 59.58%, 61.03%, 60.15%, 59.20%, 59.00%, 58.60%, 47.79%, and 48.26% sequence identity with CaCWINV1, 2, 3, 4, 5, 6, 7, CaVINV1 and 2, respectively. The predicted 3D models of CaAINVs were validated using the QMEAN server (http://swissmodel.expasy.org/qmean/cgi/index.cgi) for model quality estimation. The total QMEAN scores (estimated model reliability between 0 and 1) of the predicted 3D models for CaCWINV1, 2, 3, 4, 5, 6, 7, CaVINV1 and 2 were 0.70, 0.69, 0.71, 0.69, 0.72, 0.71, 0.66, 0.68, and 0.66, respectively. These results indicated that the predicted 3D structure of CaAINVs was reliable. CaAINVs had very similar structural models. All CaAINVs are able to form a β-propeller module at the N-terminal domain and a β-sandwich module at the C-terminal domain. Five blades (numbered I–V) assembled at the β-propeller, and the active sites formed a catalytic pocket in this module (Figure 6). In order to predict the theoretical position of sucrose when binding with MeAINVs, the primary model of CaAINVs was further structurally aligned with a model of the AtcwINV1 D239A mutant complex with sucrose (Protein Databank ID 2QQU) using the PyMOL program (Schrödinger, New York, NY, USA). The results predicted sucrose to be localized at the center of NDPNG, RDP, and WECP(V)D motifs, while the active site residues (using the numbering based on CaVINV1, the residues were D126, D250, E305, and C306) of CaAINVs were predicted to have similar orientation towards sucrose molecules (Figure 7).

### 2.6. Expression Analysis of CaAINV Genes for Different Tissues and Developmental Stages

**Figure 8:**
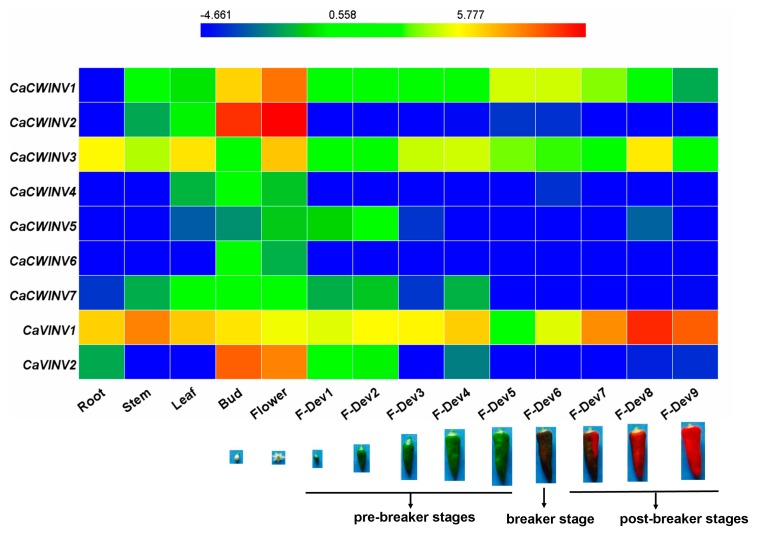
Expression profiles of nine pepper *CaAINV* genes in different tissues and developmental stages. The expression data were collected from Illumina RNA-sequence data from the genome sequences of the pepper cultivar, Zunla-1, [12]. Images of pepper fruit developmental stages were cited from Qin et al. [12]. The fragments kilobase of exon model per millon mapped reads (FPKM) values were log_2_ transformed and the heat map was generated using the HemI (Heatmap Illustrator, version 1.0) software package. The bar at the top represents log_2_-transformed values. Genes, highly or weakly expressed, in various tissues are colored blue and red, respectively.

In order to explore the expression patterns of *CaAINV* genes in pepper, RNA-sequence data from different tissues and developmental stages, such as root, stem, leaf, buds, flower, and nine stages of developing fruits, of pepper cultivar Zunla-1 [12] were used for the analysis (Figure 8). For cell wall invertase genes, *CaCWINV3,* which was expressed in all test tissues, was the highest expressed gene in the roots, stems, and leaves. *CaCWINV2* was the highest expressed gene in buds and flowers. *CaCWINV5* was mainly expressed in the F-Dev2 stage of fruits, while *CaCWINV4* and *6* was mainly expressed in buds. *CaCWINV1,* which was expressed in most test tissues, except roots, was the highest expressed gene in flowers. Of the vacuolar invertases, *CaVINV1* was expressed in all test tissues and highly expressed in mature fruits, while *CaVINV2* was mainly expressed in buds and flowers. During pepper fruit development, *CaCWINV1*, *CaCWINV3*, and *CaVINV1* were expressed in all stages. *CaCWINV1* was mainly expressed at the late pre-breaker stage (F-Dev5) and breaker stage, while *CaCWINV3* was mainly expressed at the post-breaker stage of F-Dev8. *CaVINV2* expression gradually increased during the pre-breaker stages of F-Dev1 to F-Dev4 and decreased at F-Dev5, followed by an increase at the breaker stages, and a dramatic increase at the post-breaker stages.

### 2.7. Yeast Complementation of CaCWINV-2 and CaVINV-1

**Figure 9:**
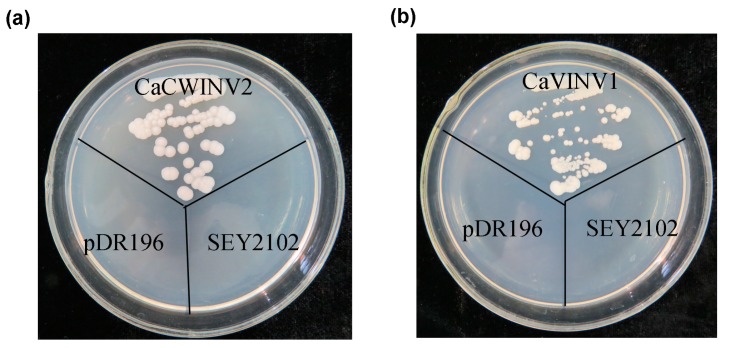
Growth of invertase-deficient strain SEY2102 transformed by pDR196-*CaCWINV2* (**a**) and pDR196-*CaVINV1* (**b**) on selective plates containing sucrose as the sole carbon source at 28 °C for 4 days. SEY2102 yeast cells transformed with empty pDR196 vector were used as control.

The temporal and spatial expression of *CaCWINV2* and *CaVINV1* indicated that they may play an important role in flower development and fruit ripening, respectively. In order to identify their catalytic effect on sucrose, the yeast triple mutant, SEY2102, which lacks endogenous invertase activity and is unable to grow on a medium with sucrose as the sole carbon source, was used for yeast complementation. The cDNA of *CaCWINV2* and *CaVINV1* were inserted into a yeast expression vector pDR196 to generate pDR196-*CaCWINV2* and pDR196-*CaVINV1*. The results showed that SEY2102 yeast cells transformed with empty pDR196 vector were unable to grow on medium containing sucrose as the sole carbon source, whereas the SEY2102 yeast cells transformed with pDR196-*CaCWINV2* or pDR196-*CaVINV1* grew on this medium (Figure 9). This result indicated that proteins encoded by *CaCWINV2* and *CaVINV1* exhibited invertase activity.

### 2.8. Optimum pH Determination for CaCWINV2 and CaVINV1

**Figure 10:**
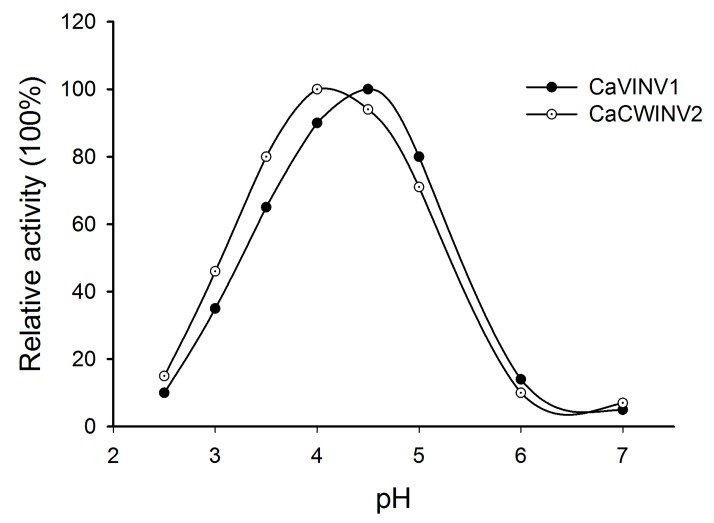
The pH dependence of the acid invertase activity of CaCWINV2 and CaVINV1.

In order to confirm whether CaCWINV2 and CaVINV1 proteins encoded acid invertases, crude proteins expressed by these genes were extracted from *S. cerevisiae* strain SEY2102. Their enzyme activities at pH values ranging from 2.5 to 7.0 were assayed. The results indicated that optimum pH for CaCWINV2 was 4.0, while the optimum pH for CaVINV1 was 4.5 (Figure 10). These results indicated that CaCWINV2 and CaVINV1 were acid invertases.

## 3. Discussion

### 3.1. Identification and Characterization of CaAINV Genes

Acid invertase activity is essential for flower and fruit development in higher plants. In such reproductive organs, CWINVs play an important role in phloem unloading, while VINVs mainly regulate cell expansion and sugar accumulation. It has been reported that VINV activity was higher than that of CWINV and sucrose synthase in all floral structures, and acid invertases activity increased with increasing hexose sugar concentration, during pepper fruit ripening [4,13]. These results indicated that acid invertase may play an important role in carbohydrate metabolism during the development of pepper flowers and fruits. However, no further information regarding the acid invertase gene family in pepper is available. In the current study, nine acid invertase genes were found in the pepper genome (Table 1). Based on subcellular localization prediction, protein sequencing, and evolutionary analysis, seven pepper acid invertases were cell wall invertases (*CaCWINV1* to *7*) while two were vacuolar invertases (*CaVINV1 to 2*). CaVINVs (70.94 to 73.29 kDa) exhibited a higher molecular weight range than the CaCWINVs (64.36 to 67.41 kDa), while CaVINVs (6.52 to 9.60) have a lower theoretical pI range than CaCWINVs (5.51 to 5.97). All of the CaAINV proteins were found to contain NDPNG, RDP, and WECP(V)D motifs, which are consistent with reported AINVs in other plants [14,15]. The second exon in all *CaAINVs* was a mini exon, located in the β-fructosidase motif (NDPNG), which encoded three amino acids. This is a typical structural feature of acid invertase genes in plants [7]. Gene pairs of *CaCWINV1*/*2* and *CaCWINV3*/*4* had similar orientation and intron-exon structures on chromosomes 9 and 10, respectively, indicating that these genes pairs were tandem repeats. Tandem duplication events found in tomato CWINV genes, *Lin5* and *Lin7*, contained the same orientation and tandem repeats localized on chromosome 9, and those found in *Lin6* and *Lin8*, had the same orientation and tandem repeats localized on chromosome 10 [16]. Evolutionary analyses showed that *CaCWINV2* and *Lin7*, *CaCWINV1* and *Lin5*, *CaCWINV3* and *Lin6*, and *CaCWINV4* and *Lin8*, respectively, formed a clade. These tandem duplications events of pepper and tomato suggest that a genomic duplication event of a progenitor cell wall invertase occurred in a common ancestor of pepper and tomato. The crystal structure of acid invertase from *Arabidopsis* (AtcwINV1) was reported [17]. The predicted 3D structural models showed that all CaAINV proteins are able to form a β-propeller module at the N-terminal domain and a β-sandwich module at the C-terminal domain. This structure is typical of plant acid invertase proteins, such as the vacuolar invertase, Bobfruct3, in bamboo [10], acid invertase in cassava [14,15], and cell wall invertase, AtcwINV1, in *Arabidopsis* [17]. The sucrose molecules were predicted to bind CaAINVs at the center of the β-propeller module, in which NDPNG, RDP, and WECP(V)D motifs and active site residues form a catalytic pocket. These structures are typical of AtcwINV1 proteins [18]. Predicted 3D structure analyses of CaAINVs suggest that all acid invertases from pepper may catalyze irreversible hydrolysis of sucrose to glucose and fructose under acidic conditions.

### 3.2. Differential Expression and Enzymatic Activities of CaAINVs

Expression patterns of *CaAINVs* in different tissues and development stages of pepper fruit may provide a basis for understanding their physiological functions. *CaAINVs* genes displayed differential expression in roots, stems, leaves, buds, flowers, and various fruit developmental stages. Reportedly, acid invertases play an important role in root formation [19], stem development [20], and delayed leaf senescence [21]. In this study, *CaCWINV3* was the highest expressed *CWINV* gene in roots, stems, and leaves, while *CaVINV1* was the highest expressed VINV gene in these tissues. This result suggested that these two genes may play a key physiological function in vegetative organs. By contrast, *CaCWINV2* was the highest expressed *CWINV* gene in buds and flowers, while *CaVINV2* was the highest expressed *VINV* gene in these tissues, indicating that these two genes play a key role in flower development by regulating sucrose metabolism. Based on evolutionary analyses, *CaCWINV1* and *Lin5*, and *CaCWINV2* and *Lin7*, respectively, formed a clade. The expression patterns of genes in each clad were similar; *CaCWINV1/Lin5* was mainly expressed in flowers and fruits, whereas *CaVINV2/Lin7* was mainly expressed in flowers [16]. *Lin7* regulates CWINV activity to increase the rate of tomato fruit set under heat stress [22], implying that *CaVINV2* may play a function similar to that of *CaCWINV2*. Lin5 activity mediates bursting during transition of ovary to fruit in tomato [23], while the expression of all *CaAINVs* down regulated this transition process. Therefore, we speculate that other sucrose metabolism related enzymes, such as sucrose synthase, may be involved in the transition from ovary to fruit in pepper. The expression of pepper vacuolar invertase gene, *CaVINV1*, increased gradually during pre-breaker stages and reached high levels during post-breaker stages. Vacuolar invertase activity plays an important role in tomato fruit ripening [24]. Our results indicated that *CaVINV1* may promote fruit development and ripening by regulating sucrose catabolism. Further investigation demonstrated that CaCWINV2 and CaVINV1 complemented an invertase-deficient yeast strain, enabling it to grow on a medium with sucrose as the sole carbon source, indicating, in turn, that CaCWINV2 and CaVINV1 may catalyze the hydrolysis of sucrose. The optimum pH of acid invertases is acidic [25]. The optimum pH for enzyme activity of CaCWINV2 was 4.0, while the optimum pH of CaVINV1 was 4.5, indicating that these proteins were acid invertases.

## 4. Materials and Methods

### 4.1. Identification and Sequence Analysis of CaAINV Proteins in Pepper

Eight AINV proteins identifiers from *Arabidopsis* were used for the initial BLASTN search against the pepper genome databases (http://peppersequence.genomics.cn/page/species/index.jsp, release 2.0) [8]. Subsequently, each identified CaAINV protein was confirmed via Pfam (http://pfam.xfam.org/search) and SMART (http://smart.embl-heidelberg.de/). Molecular weight (*M*_w_), theoretical isoelectric point (PI), and multiple alignment of CaAINV proteins were assessed using DNAman 6.0 software (Lynnon Biosoft, Quebec City, QC, Canada). Subcellular localization of CaCWINV proteins were predicted using TargetP (http://www.cbs.dtu.dk/services/TargetP/, version 1.1) and the putative signal peptides were predicted using SignalPServer (http://www.cbs.dtu.dk/services/SignalP/, version 4.1). The transmembrane domain (TMD) of CaVINV was predicted using the TMHMM Server (http://www.cbs.dtu.dk/services/TMHMM/, version 2.0). The complex N-terminal propeptide (NTPP) region of CaVINV corresponding to the residues of *Arabidopsis* vacuolar invertase AtVI2, proposed by Xiang et al., were depicted [9].

### 4.2. Phylogenetic Analyses

Phylogenetic analyses were performed on 35 CaAINV proteins from tomato, *Arabidopsis*, cassava, and poplar by comparing with the CaAINV proteins identified in this study. Multiple sequence alignments of full-length protein sequences were performed using the MUSCLE program. The phylogenetic tree was constructed via Molecular Evolutionary Genetics Analysis, Version 7.0 (MEGA7, Tokyo Metropolitan University, Tokyo, Japan) using the Neighbor-Joining (NJ) method and the bootstrap test was carried out with 1000 replicates

### 4.3. Exon–Intron Structure Analysis and Chromosomal Mapping

The exon–intron structure of the pepper *CaAINV* genes was determined by comparing cDNAs with their corresponding genomic DNA sequences from the pepper genome database (http://peppersequence.genomics.cn/page/species/index.jsp, release 2.0). Gene structures were visualized using The Gene Structure Display Server (GSDS) program (http://gsds.cbi.pku.edu.cn/, version 2.0) [26]. Chromosomal localization of the pepper *CaAINV* genes and the total length of each chromosome were drawn based on the pepper genome database. Tandem *CaAINV* gene duplications were identified as genes separated by ten or fewer gene loci at a distance range of 200 kb.

### 4.4. Conserved Motif Analysis

In order to identify the structural divergence of *AINV* genes from pepper and *Arabidopsis*, conserved motifs in the encoded proteins were analyzed using the Multiple Expectation Maximization for Motif Elicitation (MEME) online program (http://meme-suite.org/tools/meme, version 4.12.0) and visualized with TBtools. Parameters were set as follows: Distribution of motif occurrences—0 or 1 per sequence, maximum number of motifs—10; minimum motif width—6; and maximum motif width—20; all other parameters were default.

### 4.5. Prediction of Three-Dimensional Structure of the CaAINV Proteins

The three-dimensional structure of nine pepper CaAINV proteins were predicted using the Swiss-Model (http://www.swissmodel.expasy.org), and *Arabidopsis* AtcwINV1 protein structure (Protein Databank ID 2AC1, gene accession code At3g13790) was used as the template. The three-dimensional structure and NDPNG, RDP, and WECP(V)D motifs of CaAINVs were displayed using Pymol software (Delino Scientific, San Carlos, CA, USA). CaAINV models were further structurally aligned with *Arabidopsis* AtcwINV1 D239A mutant–sucrose complex (Protein Databank ID 2QQU) using Pymol software to predict the theoretical position of sucrose when binding to CaAINVs.

### 4.6. Analysis of Expression Characteristics of CaAINV Genes

The expression profiling of pepper *CaAINV* genes in different tissues, such as roots, stems, leaves, buds, and flowers, and the nine fruit developmental stages, was obtained from the RNA-sequence data of Zunla-1 pepper cultivar [12]. Fragments per kilobase of transcript per million fragments mapped (FPKM) was used to represent *CaAINV* expression levels. Log_2_-transformed RPKM values of *CaAINV* were used to draw a heat map via HemI (Heatmap Illustrator, version 1.0, Huazhong University, Wuhan, China) software packages.

### 4.7. Yeast Complementation and Enzymatic Analysis of CaCWINV2 and CaVINV1

Full-length cDNAs of the *CaCWINV2* and *CaVINV1* were isolated from flowers of Zunla-1 pepper cultivar by RT-PCR. The cDNA of these genes were sequenced by Sangon Biological Engineering Technology and Services (Shanghai, China). To confirm the function of *CaCWINV2* and *CaVINV1*, an invertase-deficient strain SEY2102 was used to perform *S. cerevisiae* complementation assays [27]. The coding sequences of *CaCWINV2* and *CaVINV1* were inserted into the yeast shuttle vector pDR196, containing *URA3* as a selective marker, respectively. The new plasmids were confirmed via sequencing and named pDR196-*CaCWINV2* and pDR196-*CaVINV1*. These new plasmids and the empty vector, pDR196, were transformed into the SEY2102 strain using the PEG/LiAc method, and the transformants were selected on synthetic dropout (SD) medium without uracil. The catalytic function of CaCWINV2 and CaVINV1 was determined by the growth status of the transformant strain on SD medium, with sucrose as the sole carbon source. The SEY2102 yeast cells, transformed with pDR196, pDR196-CaCWINV2, and pDR196-CaVINV1, were grown in 30 mL of SD liquid medium (−URA) for 3 days. Yeast cells were harvested by centrifugation and yeast proteins were extracted for enzyme activity analysis. Analysis of the enzyme activities of CaCWINV2 and CaVINV1 at different pH values was carried out as per the method described by Liu et al. [27].

## Figures and Tables

**Table 1:** Basic information of seven pepper alkaline/neutral invertase genes (*CaAINVs*).

Gene Name	Gene ID	ORF Length (bp)	Protein Length (aa)	*M*_w_ (kDa)	PI	Localization
*CaCWINV1*	Capana09g002436	1773	590	67.41	9.23	Cell wall
*CaCWINV2*	Capana09g002437	1743	580	65.86	9.07	Cell wall
*CaCWINV3*	Capana10g002008	1746	581	65.78	9.60	Cell wall
*CaCWINV4*	Capana10g002007	1773	590	66.80	9.09	Cell wall
*CaCWINV5*	Capana10g001560	1737	578	66.16	9.16	Cell wall
*CaCWINV6*	Capana00g001535	1734	577	66.17	6.92	Cell wall
*CaCWINV7*	Capana03g000156	1719	572	64.36	6.52	Cell wall
*CaVINV1*	Capana03g002552	1932	643	70.94	5.97	Vacuole
*CaVINV2*	Capana01g000522	1971	656	73.29	5.51	Vacuole
